# From Green Space to Green Prescriptions: Challenges and Opportunities for Research and Practice

**DOI:** 10.3389/fpsyg.2017.00268

**Published:** 2017-02-27

**Authors:** Agnes E. Van den Berg

**Affiliations:** Department of Cultural Geography, Faculty of Spatial Sciences, University of GroningenGroningen, Netherlands

**Keywords:** care farming, fractals, green care, green exercise, healing environment, nature-based therapies, randomized controlled trial, restorative environment

## Green space and health

Research on the healthy and restorative effects of green space has been rapidly expanding over the past decades, and the field is fast moving toward maturity (van den Berg and van den Berg, 2014; Capaldi et al., 2015). Findings converge in showing that regular contact with green space can enhance well-being and alleviate stress, and may even mitigate income-related health inequalities regarding chronic diseases and life expectancy (Mitchell and Popham, 2008). In response to these insights, there has been a surge of initiatives to (re)connect people with nature, especially those without the ability or opportunity to engage with green space as part of their usual lifestyle. Many of these initiatives have focused on “bringing nature to people” by greening of places in people's nearby environment, such as schoolyards, urban public spaces, hospitals, classrooms, and offices (Wolch et al., 2014; van den Berg et al., 2016). Other initiatives have aimed at “bringing people to nature” by encouraging and facilitating adults and children to actively participate in nature-based activities (Bragg and Atkins, 2016). These activities range from health promotion programs and projects for the general population, like green gyms or community gardening, to more therapeutic interventions for individuals with a defined need, like care farms, walk-and-talk coaching, or horticultural therapy.

Nature-based activities are increasingly gaining momentum as a cost-effective, easy-to-do, low-risk, and enjoyable preventive and therapeutic intervention that is particularly beneficial for people who do not have the ability or opportunity to engage with nature as part of their usual lifestyle (Allen and Balfour, 2014). For example, a calculation of the social return on investment of a health walks program in Glasgow revealed a cost-benefit ratio of £8 for every £1 invested (Carrick, 2013). It therefore seems important that this type of intervention becomes more routinely integrated into everyday health care practice. In particular, general practitioners and other primary health professionals have been targeted as key actors who can support such integration by writing more “green prescriptions” (Hine et al., 2008; Patel et al., 2011; Nisbet and Lem, 2015). However, although objective data are scarce, the overall impression in most countries is that referral rates tend to be very low (Maas and Verheij, 2007; Allen and Balfour, 2014). Therefore, a key question is: what is needed to make health professionals write more green prescriptions?

## Challenges for the health care sector

The low rates of green prescriptions may at least be partly due to the complexity of translating innovations into health care practice, which often requires changes on the side of both the providers of the innovative services and the health professionals who refer patients to these services (Fleuren et al., 2004). Although, each country has its unique context, some common challenges for providers of nature-based interventions can be identified that transcend national boundaries (Bragg and Atkins, 2016). These challenges include (a) the development of a consistent terminology and common language to describe the field, (b) better collaboration between providers and streamlining of communication to health professionals, (c) professionalization of the services by developing quality standards and tools for monitoring and evaluation of quality and effectiveness, and (d) improvement of access to services to health professionals, for example, by registration in service directories and inclusion in clinical guidelines.

Regarding the development of a common language, there is an increasingly felt need for a concise and recognizable umbrella term to promote the full range of preventive and therapeutic health interventions and programs that make use of elements of nature. “Green exercise” has recently been propagated as a general concept that “implies a synergistic health benefit of being active in the presence of nature” (Barton et al., 2016). However, the term is not suitable as an overarching term because it reduces green space to a supportive environment for exercise, thereby ignoring the direct beneficial effects of being immersed in green. “Green care” has been broadly defined as “utilizing plants, animals, and landscapes to create interventions to promote health and well-being” (Sempik and Bragg, 2013), and thus seems to offer promise as a sufficiently broad term of choice. Contrary to this notion, however, the UK Green Care Coalition has recently proposed to restrict the use of “green care” to interventions aimed at people with a defined or diagnosed need (Bragg and Atkins, 2016). This restriction seems artificial and confusing, given that many nature-based interventions (like health walks or gardening programs) are applied in a similar manner to specific as well as general populations. Instead of narrowing the definition, “green care” could be viewed more broadly to include all organized nature-based health interventions and programs for defined and general populations. More fine-grained distinctions can be expressed by variations like “green therapeutic care” or “green community care.”

While providers and health professionals are the primary actors when it comes to the integration of “green care” (defined in a broad manner) in medical practice, environmental psychologists and other researchers within the green space-health domain also have a role to play. The legitimacy of green care strongly depends on the scientific evidence for a relationship between green space and health. The scientific research agenda therefore has important and far-reaching implications for the acceptance of green care and the implementation of green prescriptions.

## The importance of clinical evidence

Research on beneficial effects of contact with green space has been dominated by epidemiological studies linking green space to public health, and experimental studies on the stress-relieving and mood-enhancing effects of interacting with green space (see for an overview, van den Berg and van den Berg, 2014). The latter, so-called “restorative environments studies,” show many similarities with randomized controlled trials (RCTs), such as random assignment of participants to experimental and control conditions and the use of validated outcome measures (Friedman et al., 2010). However, restorative environment studies also differ from RCTs in important ways. Most studies focus on brief exposure to (simulated) natural environments among healthy individuals; only few randomized controlled studies have examined more long-term intervention programs and therapies among patient samples (see for reviews, Annerstedt and Währborg, 2011; Clatworthy et al., 2013; Kamioka et al., 2014). Furthermore, restorative environment studies typically do not follow the strict guidelines for good clinical practice, like pre-trial registration and the use of standardized protocols. Thus, although there is a strong experimental tradition in green space—health research, most of the experiments do not comply with strict criteria for RCTs, and do not qualify as clinical evidence.

Health professionals widely believe that only RCTs can produce trustworthy results (Concato et al., 2000). Moreover, RCTs can provide information on optimal dosing, treatment duration, and effectiveness of different types of interventions for different groups. This would seem to suggest a straightforward recommendation for more RCTs. However, a practical issue in applying RCTs to nature-based interventions to is that patients and administrators cannot be easily blinded to the intervention: it is usually very noticeable that the treatment takes place in natural surroundings, instead of the standard care environment. If blinding is not possible, negative or positive views of the intervention by patients or administrators may be a major source of bias, and the outcomes may not be taken as seriously as the outcomes of (double) blinded trials. Thus, although the availability of RCTs is a key requirement for a greater acceptance by health professionals, RCTs do not lend themselves well to nature-based interventions, and therefore are unlikely to be sufficient in convincing health professionals to write more green prescriptions.

## The need for a convincing explanatory framework

Besides a strong clinical evidence base, an important prerequisite for the acceptance of green care is a convincing explanatory framework that specifies the main pathways and causal mechanisms of these interventions. Since the natural environment is a central component, an explanatory framework for green care should specifically elucidate how interacting with nature and green space may promote health. The available evidence suggests three main pathways: (a) regulation of immunological and physiological (stress) responses, (b) enhancement of psychological states like mood, self-esteem, vitality, and attention, and (c) facilitation of health-promoting behaviors such as exercise and social contacts. In a recent review, Kuo (2015) proposed that most known pathways between green space and health can be subsumed under the central biological pathway of enhanced immune functioning. This is an important new insight that, if substantiated, would provide a plausible medical explanation that may increase adoption of green care by health professionals.

Researchers have also worked toward unraveling the causal mechanisms involved in the direct physiological and psychological effects of green space. Traditionally, two theories have been invoked to explain these effects. First, stress-reduction theory holds that natural settings evoke an “automatic positive affective response,” which blocks negative thoughts and feelings and reduces physiological activation (Ulrich et al., 1991). Second, attention restoration theory proposes that natural settings evoke “soft fascination,” capturing attention in a pleasant, effortless bottom-up manner, without taxing executive processes (Kaplan, 1995). What remains unclear in both theories, however, is precisely which environmental cues trigger the “automatic positive affective response” and “soft fascination” (Valtchanov and Ellard, 2015). In other words, if green space is a medicine, then what are its active ingredients?

## Potential active ingredients of green space as medicine

Using advanced image decomposition techniques, recent research has shown that positive responses to viewing nature scenes images are causally related to low level spatial and color features of the scenes (Kardan et al., 2015; Valtchanov and Ellard, 2015). Of these low level features, particularly fractals have gained much interest (Joye and van den Berg, 2011). Fractals are self-similar patterns that can be found throughout the natural world, but are uncommon in human-made structures (Mandelbrot, 1983). Among other things, it has been demonstrated that viewing computer-generated natural fractals can increase EEG-recorded alpha waves, an indicator of a wakefully relaxed state (Hägerhäll et al., 2008, 2015).

People's interactions with nature typically involve a full, multisensory experience, instead of mere visual exposure. A convincing explanatory framework would therefore include not only visual, but also other types of sensory cues. In this respect, olfactory cues, such as phytoncides, have recently come into focus (Craig et al., 2016). Phytoncides are chemical substances with antibacterial and fungal qualities that are secreted by many plants and trees to protect themselves. Preliminary findings indicate that inhaling phytoncides can evoke physiological and psychological responses in humans similar to the responses evoked by a visit to nature and green space (Li et al., 2006).

The recent advances in basic research on fractals and other low level features of natural environments mark an important turning-point in the understanding of health benefits of green space and green care, because they speak against the idea that these benefits are merely a “placebo effect,” produced by people's culturally and personally shaped expectations of what nature and green space can do for them. It is therefore important that researchers continue to explore this line of work. However, to avoid the pitfalls of reductionism, other more holistic and experiential perspectives should also be taken into consideration. In particular, the concept of “connectedness to nature” has recently come to the fore as a mechanism that strikes a chord with many who have personally experienced the benefits of interacting with green space (Capaldi et al., 2015).

## Conclusion

In the past decades, research on the health benefits of green space has made great strides, which has led many countries to actively work toward implementing nature-based interventions that make use of the preventive and therapeutic potential of green space. However, health care professionals are still reluctant in prescribing these interventions to their patients. To sway these professionals, providers need to agree on a common language for describing their services, and researchers need to give more attention to clinical trials as well as to basic research on the biological pathways and explanatory mechanisms. With the help of these developments, a transition can be made from green space to green prescriptions.

## Author contributions

The author confirms being the sole contributor of this work and approved it for publication.

### Conflict of interest statement

The author declares that the research was conducted in the absence of any commercial or financial relationships that could be construed as a potential conflict of interest.
